# Factors associated with health-related quality of life among home-dwelling older adults aged 75 or older in Switzerland: a cross-sectional study

**DOI:** 10.1186/s12955-022-02080-z

**Published:** 2022-12-21

**Authors:** Flaka Siqeca, Olivia Yip, Maria José Mendieta, Matthias Schwenkglenks, Andreas Zeller, Sabina De Geest, Franziska Zúñiga, Samuel Stenz, Matthias Briel, Carlos Quinto, Eva Blozik, Mieke Deschodt, Katrina Obas, Suzanne Dhaini

**Affiliations:** 1 Department of Public Health, Institute of Nursing Science, University of Basel, 4051 Basel, Switzerland; 2 Department of Public Health and Primary Care, Academic Centre for Nursing and Midwifery, KU Leuven, 3000 Leuven, Belgium; 3 Department of Public Health, Institute of Pharmaceutical Medicine (ECPM), University of Basel, 4051 Basel, Switzerland; 4 Department of Clinical Research, Center for Primary Health Care, University of Basel, 4051 Basel, Switzerland; 5 Department of Clinical Research, Division of Clinical Epidemiology, University Hospital Basel and University of Basel, 4051 Basel, Switzerland; 6 Department of Health Research Methods, Evidence, and Impact, McMaster University, Hamilton, Canada; 7 Aerztegesellschaft Baselland, 4132 Muttenz, Switzerland; 8 Helsana-Gruppe, 8001 Zurich, Switzerland; 9 Institute of Primary Care, University of Zurich and University Hospital of Zurich, 8091 Zurich, Switzerland; 10 Department of Public Health and Primary Care, Gerontology and Geriatrics, KU Leuven, 3000 Leuven, Belgium; 11 Competence Center of Nursing, University Hospitals Leuven, Leuven, Belgium; 12 Department of Epidemiology and Public Health, Swiss Tropical and Public Health Institute, 4051 Basel, Switzerland

**Keywords:** Health related quality of life, Quality of life, EQ-5D, Healthy aging, Ecological model, Demographic survey, Home-dwelling older adults

## Abstract

**Background:**

HRQoL is an indicator of individuals’ perception of their overall health, including social and environmental aspects. As a multidimensional concept, HRQoL can be influenced by a multitude of factors. Studies of HRQoL and factors associated with it among home-dwelling older adults have often been limited to inpatient settings or to a sub-population with a chronic disease. Studying HRQoL and its correlating factors among this population, by providing an ecological lens on factors beyond the individual level, can provide a better understanding of the construct and the role of the environment on how they perceive their HRQoL. Thus, we aimed to assess the HRQoL and investigate the correlates of HRQOL among home-dwelling older adults, guided by the levels of the ecological model.

**Methods:**

This is a cross-sectional population survey conducted in 2019 in Canton Basel-Landschaft, in northwestern Switzerland, and includes a sample of 8786 home-dwelling older adults aged 75 and above. We assessed HRQoL by using the EQ-index and the EQ-VAS. The influence of independent variables at the macro, meso and micro level on HRQoL was tested using Tobit multiple linear regression modelling.

**Results:**

We found that having a better socio-economic status as denoted by higher income, having supplementary insurance and a higher level of education were all associated with a better HRQoL among home-dwelling older adults. Furthermore, being engaged in social activities was also related to an improved HRQoL. On the other hand, older age, female gender, presence of multimorbidity and polypharmacy as well as social isolation and loneliness were found to all have a negative impact on HRQoL.

**Conclusions:**

Understanding factors related to HRQoL by using an ecological lens can help identify factors beyond the individual level that impact the HRQoL of home-dwelling older adults. Our study emphasises the importance of social determinants of health and potential disparities that exists, encouraging policymakers to focus on policies to reduce socio-economic disparities using a life-course approach, which consequently could also impact HRQoL in later stages of life.

**Supplementary Information:**

The online version contains supplementary material available at 10.1186/s12955-022-02080-z.

## Background

Many European countries have experienced an increase in the number of people living longer. In 2021, 20.8% of the European population was 65 years or older and 6.0% was 80 years or older, a proportion projected to continue rising [1]. Demographic data in Switzerland depicts a similar picture, where between 2020 and 2050, an increase from 18.0 to 25.6% for those 65 years or older and from 5.0 to 10.6% for those 80 years and older is predicted [2]. To cope with this demographic shift, research and policy actions have changed focus to support older adults to continue living in the community instead of relying on long-term institutions [3]. This is also favored by older adults themselves, who prefer to age in their own home and familiar environment for as long as possible [4, 5], an objective described by Cutchin et al. as ‘aging in place’ [6]. Aging in place has been shown to positively affect the quality of life of older adults [7, 8] as it fosters preservation of their autonomy and social connectiveness [9].

Quality of life is defined by the WHO as “individuals' perceptions of their position in life in the context of the culture and value systems in which they live and in relation to their goals, expectations, standards and concerns” [10]. It is a broad concept that incorporates all aspects of an individual's existence whereas health-related quality of life (HRQoL) focuses on the health-related aspects of quality of life,  including people’s level of daily functioning and ability to experience a fulfilling life [11]. However, it is important to note that the terms are not interchangeable [12]. HRQoL is a key patient-reported outcome and an indicator of an individual’s perception of their overall health, be that physical, functional, emotional, or mental; and includes the influence of the social determinants of health such as receiving support from family and community as well as being active in the society [13]. During the past decades, several generic measures of HRQoL have been developed, such as the Short-Form 6-dimensions (SF-6D) [14], the Health Utilities Index (HUI) system [15] and the EuroQoL 5-dimensions questionnaire (EQ-5D) [16]. In this study, we used the EQ-5D-5L instrument, which is a simple, robust, reliable, and user-friendly instrument, that takes short time for respondents [17]. It is an instrument constructed for use as a general measure of HRQoL, and has been increasingly used in research in older adults [18–21].

HRQoL is a multidimensional concept and can thus be influenced by a myriad of factors. In older adults, sociodemographic factors such as advanced age; lower education and income; as well as the presence of chronic diseases, smoking, depression, and lack of social support were all found to be associated with a lower HRQoL [22–29]. Furthermore, when exploring perceptions and lived experience of home-dwelling older adults in relation to their HRQoL, Levasseur et al. identified that for older adults, having a social role and engaging in social activities also played an important part in determining their perceived HRQoL as better [30].

Despite the fact that HRQoL has been widely investigated in older age in terms of factors associated with it, to the best of our knowledge most studies have focused on assessing it in inpatient settings and in relation to a specific disease or chronic condition [31–34]. We believe that investigating HRQoL among home-dwelling older adults in the community, while taking into account their ecosystem through an ecological perspective, has hence been overlooked. HRQoL and the factors associated with it are of interest to be studied among this population to not only foster individual well-being but also shape policies and strategies aimed at preserving the autonomy and social relations of older adults living in the community.

To support older adults to continue living in the community, we launched the INSPIRE project, which is an implementation science project. The project aims to develop, implement and evaluate a community-based integrated care model for home-dwelling older adults aged 75 and above in Canton Basel-Landschaft (BL) in Switzerland. During the development phase, an understanding of the context to ensure suitability of the integrated care model components for the implementation setting was pivotal [35, 36]. Accordingly, we conducted the INSPIRE Population Survey to understand current and anticipated health and social needs as well as living preferences, in an effort to maintain HRQoL and support older adults to age in place [37]. Aging in place has been shown to positively affect the HRQoL of older adults, as it fosters preservation of their autonomy and social connectiveness, and is the reason why we aimed to assess the current HRQoL and what factors influence this construct, using an ecological approach.

### Conceptual model

**Fig. 1:**
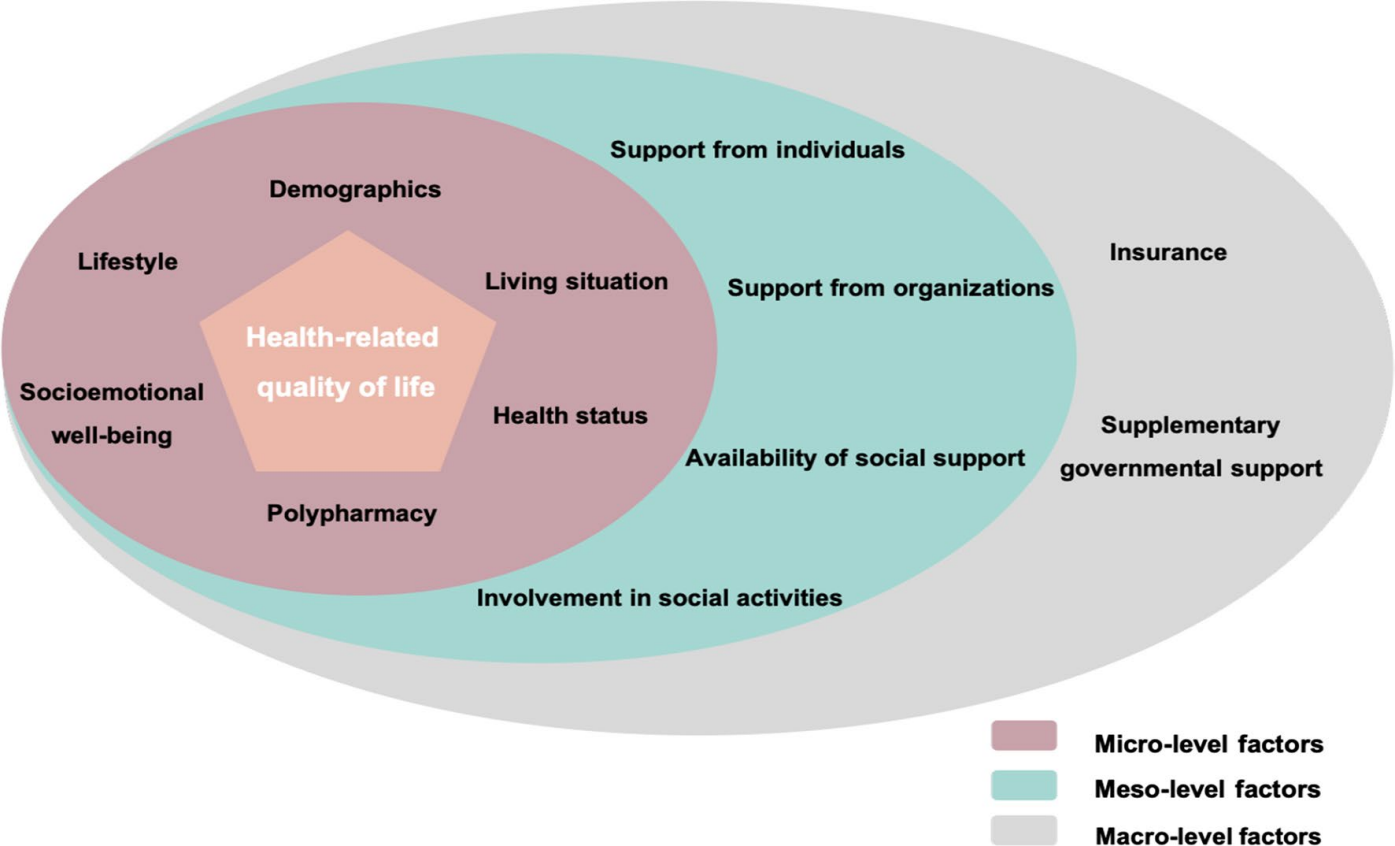
Health-related quality of life conceptual model (guided by levels of the ecological model)

As HRQoL is a multidimensional construct, using an ecological approach can provide a comprehensive understanding of the variables at the micro, meso and macro level that are associated with it. An ecological approach is founded on the idea that a dynamic interrelationship exists among various correlates at multiple levels including personal (i.e., biological, psychological), organizational/institutional, environmental (i.e., social and physical) and policy levels [38]. Our proposed conceptual model is not explicitly based on a specific pre-existing framework, but instead draws from current literature on factors influencing HRQoL among older adults. The model places the older adults and their perceived HRQoL in the center, while enlisting the potential correlating variables from literature in the three levels of the ecological model (micro, meso and macro level) (Fig. 1).

The overall objectives of this paper are to (1) assess the HRQoL among home-dwelling older adults aged 75 and above and (2) investigate the correlates of HRQOL in this population, guided by the levels of the ecological model.

## Methods

### Study design and setting

This is a cross-sectional study conducted in 2019 in Canton BL, in northwestern Switzerland [37]. Canton BL is a German-speaking region, inhabited by around 290,000 citizens and has the second-highest proportion of population aged 65 or above (22.4%) and aged 80 or above (6.7%) in Switzerland [2].

### Study participants and data collection

The participants were recruited via postal mail, with no sampling method necessary as we included all those eligible, namely all home-dwelling older adults living in Canton BL who were aged 75 and above. The INSPIRE Population Survey is embedded within the larger INSPIRE project (https://inspire-bl.unibas.ch/), in which an important component of the care model is screening for frailty. As frailty increases with age [39], the age cut-off of 75 years was chosen as an age when we consider older adults are more likely to be at risk of frailty and can thus benefit the most from the integrated care intervention.

A survey package containing the questionnaire along with instructions for filling it out, an information sheet, a personalized cover letter, a prepaid return envelope and the informed consent form was mailed to the home address of all community-dwelling persons aged 75 years or older in Canton BL, which we received from the Cantonal Statistical Office. Thus, the filled-out questionnaires were also returned by postal mail. All the questionnaires were pseudonymized prior to being delivered, with the intent to allow potential follow-up in the future. However, due to concerns of the general public on data security and based on several stakeholder recommendations, we anonymized the questionnaires after having sent them and destroyed all documents containing identifiable information.

The survey was successfully delivered to 28,791 older adults living at home in Canton BL and a total of 8,846 questionnaires were returned (Response Rate = 30.7%). During the validation process, 60 questionnaires were excluded from the analysis (i.e., based on ineligible ZIP codes, respondent’s age, or residents in a long-term care institution), resulting in a final sample of 8786 participants. We consider the response rate to be representative, as it is much higher than what is reported in literature for postal surveys [40]. Furthermore, we found that the prevalence of frailty among community-dwelling older adults as measured by the GFI in a comparable study population to be in line with our observed results [41].

A detailed description on the development, dissemination and characteristics of the population survey have been reported elsewhere [37].

### Variables and measurements

As the current study is part of an implementation science project, the survey was designed with the input of various stakeholders. The list of stakeholders includes but is not limited to a group of older adults, representatives of local policymakers, community care providers and representatives of nursing homes. The survey items are henceforth a combination of validated tools and investigator-developed items. Detailed information on the development of the survey and overall participants’ characteristics have been reported elsewhere [37].

#### Outcome variable

*HRQoL* was assessed using the EQ-5D-5L instrument [16], a generic standardized instrument comprising of a short descriptive questionnaire and a visual analogue scale (EQ-VAS). The descriptive questionnaire includes the following dimensions of health: *mobility, self-care, usual activities, pain/discomfort and anxiety/depression*. Each dimension has a five-level response of severity, ranging from 1—no problems, 2—slight problems, 3—moderate problems, 4—severe problems to 5—unable to/extreme problems, which correspond to potential health states [42]. These health states are then converted into a single EQ-5D-index, by applying a country-specific valuation algorithm [42]. In lack of a Swiss value set, we used the German value set algorithm by Ludwig et al. [43]. Along with the descriptive questionnaire, this instrument also includes a visual analogue scale (EQ-VAS). This scale is similar to a thermometer, where the endpoints are labelled ‘The worst health you can imagine (0)’ and ‘The best health you can imagine (100)’. The construct validity of the EQ-5D-5L instrument is well examined in use among older adults, such as in the Bhaduri et al. study, who computed the Spearman’s rho between each of the EQ-5D items and the Barthel Index (Spearman coefficients 0.42) [44].

#### Micro level variables

Year of birth was used to calculate the *age* of the participants at the time of the survey completion and was recorded as a continuous variable. *Gender* information was collected as “Male” or “Female”. The original answers categories for the *level of education* question were regrouped into four categories: “Tertiary” (“University” and “University of Applied Sciences”); “Secondary” (“Gymnasium” and “Apprenticeship”); “Elementary or None” (“Elementary School” and “No degree") and “Other”. Income, which was originally collected as a monthly household income in Swiss Francs (CHF), was converted to *individual income* by dividing the household income by the number of people living in the household, following the guideline of the Swiss Centre of Expertise in the Social Sciences on how to measure income in surveys [45].

*Living situation* of the participants was assessed through an investigator-developed item asking who they currently lived with. For the purpose of the analysis, the answer choices were dichotomized into: living alone vs living with others (a spouse/partner, an adult child, other adults, siblings or a professional caretaker).

The *health status* of the participants was assessed by asking whether they experienced *vision, hearing or memory problems* in their daily life, or if they had *unintentionally lost weight* in the past 6 months. *Polypharmacy*, defined by the Groningen Frailty Index (GFI) tool [46] as taking four or more medications at once, was also recorded. Variables pertaining to health status and polypharmacy had dichotomized “Yes” or “No” answer choices. The criterion validity of the GFI tool among older adults has been examined (r − 0.62) [47].

*Socioemotional well-being* of the participants was assessed using three questions from the GFI tool [46] which ask the participants whether they feel empty, miss the company of others or feel abandoned. The answer choices for these questions included: “Yes”, “Sometimes” or “No”, which for the purpose of the analysis were dichotomized into “Yes / Sometimes” and “No”.

The lifestyle section included questions on *smoking, alcohol intake* and *physical activity*. The participants were asked about their *smoking* habits, with answer choices being regrouped into “No” (“Not currently, but I was a smoker before” and “No”) and “Yes” (“Yes, daily” and “Yes, not daily”). Additionally, *alcohol intake* was assessed by asking the number of drinks a participant consumed in a typical day; with a glass of wine, one dosage of beer of 355 ml or a 40 ml spirit alcohol counting as one drink [48]. The answer choices for this question included: “No drink”, “1–2 drinks”, “3–4 drinks” and “5 or more drinks”. The answer choices were dichotomized into “ ≤ 2 drinks/day” or “ > 2 drinks/day”, based on recommendations of the Swiss Federal Commission for Issues Related to Addiction and Prevention [49]. The participants were also asked about how many minutes they engaged in *vigorous-intensity, moderate-intensity physical activity* and in *muscle-strengthening activities* in a typical week. The WHO recommends that an older adult should engage in at least 75 min of vigorous-intensity, or in at least 150 min of moderate-intensity physical activities within a typical week [50]. For additional health benefits, the WHO recommends that an older adult engages in muscle strengthening physical activity in at least 2 days per week [50]. Due to potential multicollinearity among these three variables, we computed one variable related to physical activity. If a person scored 1 or above, which indicated they engaged in any of the three activities as recommended, it was recorded as being physically active. The answer choices were thus scored as: “Per WHO recommendations” and “Below WHO recommendations”.

#### Meso level variables

*Informal daily support from individuals* was also assessed and answer choices were dichotomized into: currently receive support from another individual (spouse, younger family member, friend or neighbour) or currently do not need such support. Participants were also asked whether they currently received daily *support from organizations*, through listing common organizations that older adults receive support from in Switzerland. These include home care organizations, social care organizations, humanitarian organizations (e.g., Red Cross) and disease-specific associations (i.e., Diabetes association, Alzheimer`s association and Parkinson’s association). The answer choices for this question were dichotomous “Yes” or “No”.

*Availability of social support* was assessed through the Brief Social Support Scale (BS6), which has been validated in German [51]. This instrument includes three questions to assess the availability of tangible support (i.e., someone to accompany them to doctor`s appointments, someone to prepare their meals when unable to and someone to help with daily chores when sick) and three others to assess the availability of emotional support (i.e., someone who can give them good advice, someone they can confide in during a crisis and someone who understands their problems) [51]. The responses are scored on a 4-point Likert scale ranging from 1- “never” to 4- “always”. A sum score of the six-items, ranging from 1 to 24, is calculated and then dichotomized into: “Low to moderate support (a score of up to 17)” and “High to very high support (score of 18 and higher)” [51]. Reliability of the subscales has also been proven, as indicated by Cronbach´s alpha: emotional support *α* = 0.87, tangible support *α* = 0.86 and overall *α* = 0.86 [51].

To assess involvement in *social activities*, the questionnaire included an investigator-developed list of hobbies and activities (e.g., sports, political parties, church gatherings, volunteering, meeting with family and friends) for which participants could indicate whether they were active in or wished to be active in. To provide more granularity in the results, we grouped the participants into three groups: those who were active in more than one of the activities, those who were active in only one, and those who wished to be active in at least one of the listed activities, but were not currently.

#### Macro level variables

*Type of insurance* of the participants was assessed by asking them whether they were insured with statutory health insurance alone or with statutory health insurance plus supplementary private insurance. Although health insurance can be considered an individual factors as well, we have included it as a macro-level factor because in Switzerland, basic health insurance is mandatory. The benefit package of the basic insurance is more comprehensive than in most other countries and defined at the national level, where payment mechanisms are largely defined by federal and cantonal regulations.

Information on *supplementary government support* was captured by asking the participants whether they received this type of support or not. Supplementary government support is a specific type of help in Switzerland, that support individuals financially if their pension or income do not cover minimum living costs.

#### Statistical analysis

General descriptive statistics were computed for the EQ-5D-5L domains and all independent variables. Categorical variables (e.g., gender, education, etc.) are reported as frequencies and percentages whereas continuous variables (e.g., age and income) are reported as medians and interquartile ranges or means and standard deviations. The EQ-5D-5L descriptive results are presented by recording the number and percentage of patients reporting each severity level of each dimension of the EQ-5D-5L instrument.

To gain an initial understanding of the association of the independent variables with HRQoL (for both the EQ-VAS and the EQ-5D-index), standard univariate tests such as Mann–Whitney U test and the Kruskal–Wallis test were used for categorical variables. The Spearman`s correlation coefficient was used to test the association of the outcome with continuous predictors.

The influence of independent variables at the macro, meso and micro level on both EQ-5D-index and EQ-VAS were tested using multiple linear regression modelling. All covariates of the conceptual model, from all levels, were included in the regression model, irrespective of significance, in order to determine the relationships of each variable with the outcome variable. Because ceiling effects were observed in previous studies using the EQ-5D-5L in general population surveys [52], we used Tobit-regression modelling. This is a variation of multiple regression, which is capable of correct inference in the presence of ceiling effects [53]. We tested if the underlying assumptions of the linear modelling were met and used the Variance Inflation Factor (VIF) to test the presence of multicollinearity among independent variables. The level of significance was set at 0.05.

Data was primarily missing due to item nonresponse, and after the analysis of missing patterns, we considered our data to be missing at random (MAR). In our dataset, we observed two variables with more than 5% of missing data: individual income (5.3%) and availability of social support (26.6%). As our data met the recommendations of Jakobsen et al. [54] for when to use multiple imputation (i.e. missing data is above 5% but below 40%, data was missing not only on the dependent variable, the Missing Completely at Random—MCAR assumption could not be plausible, and data is considered MAR), we applied multiple imputation by chained equations (MICE) to impute missing values [55]. We also ran a sensitivity analysis using the observed data and found no significant differences in results between the observed and the imputed data.

All analyses were performed using R, version 1.3.1093 for Mac OS [56].

## Results

### Health-related quality of life descriptive results

Table 1 presents the EQ-5D-5L descriptive results by recording the number and percentage of older adults reporting each severity level of each dimension of the EQ-5D-5L instrument. The mean score of EQ-VAS was 75.2 (SD = 15.9, range 0–100; skewness − 0.98) whilst the mean score of EQ-5D-index was 0.9 (SD = 0.13, range − 0.66 to 1; skewness − 3.33).

**Table 1:** EQ-5D-5L frequencies and proportions by dimension and level

Response level	Dimension				
	Mobility n (%)	Self-care n (%)	Daily activities n (%)	Pain/discomfort n (%)	Anxiety/depression n (%)
No problems	6172 (70.7)	8093 (92.5)	6973 (79.9)	3090 (35.6)	6251 (71.8)
Slight problems	1576 (18.1)	423 (4.8)	1187 (13.6)	3682 (42.4)	1864 (21.7)
Moderate problems	759 (8.7)	151 (1.7)	384 (4.4)	1605 (18.5)	414 (4.8)
Severe problems	191 (2.2)	48 (0.5)	99 (1.1)	278 (3.2)	53 (0.6)
Extreme problems	28 (0.3)	37 (0.4)	86 (1.0)	25 (0.3)	8 (0.1)
Total (%)	8726 (99.3)	8752 (99.6)	8729 (99.4)	8680 (98.8)	8590 (97.8)

### Descriptive results of factors associated with HRQoL by levels of the ecological model

#### Micro level variables

The mean age of participants was 81.8 (SD = 4.8) and 51.8% were female. 24.6% of the participants had a tertiary education, and the mean individual income was CHF 4569 (SD = 1886) per month.

Of the 8786 participants, 23.6% stated feeling empty or sometimes feeling empty, a higher percentage (35.7%) stated to miss or sometimes miss the company of others whereas 10.6% stated feeling abandoned or sometimes feeling abandoned. Almost half of them (47.6%) reported polypharmacy, while the highest proportion in health problems was reported for memory problems (19.4%) (Table 2).

**Table 2:** Participant characteristics per levels of the ecological model

Variables	Median (IQR)/n (%) N = 8786
**Micro level variables**	
Age (in years)	81.0 (7)
Gender (Female)	4552 (51.8)
Education	
*Tertiary*	2159 (24.6)
*Secondary*	4854 (55.2)
*Primary/No education*	1376 (15.6)
*Other*	397 (4.5)
Income (in CHF)	4500 (1628)
Living situation	
*Living alone*	3161 (36.0)
*Living with others*	5625 (64.0)
Reported vision problems	783 (8.9)
Reported hearing problems	1570 (17.9)
Reported memory problems	1713 (19.4)
Reported unintentional weight loss in past 6 months	379 (4.3)
Reported polypharmacy	4184 (47.6)
Reported feeling empty / sometimes feeling empty	2079 (23.6)
Reported to miss company / sometimes miss company	3134 (35.7)
Reported feeling abandoned / sometimes feeling abandoned	936 (10.6)
Physical activity	
*As per recommendations of WHO*	6895 (78.4)
*Below the recommendations of WHO*	1891 (21.5)
Alcohol intake	
*≤ 2 drinks/day*	8187 (93.1)
*> 2 drinks/day*	599 (6.9)
Reported to be currently smoking	611 (6.9)
**Meso level variables**	
Receive support from individuals	3204 (36.4)
Receive support from organizations	2688 (30.6)
Availability of social support	
*High to very high*	1727 (19.7)
*Low to moderate*	7059 (80.3)
Social activities	
*Active in more than one activity*	4382 (49.9)
*Active in one activity*	3145 (35.8)
*Not currently active / wish to be*	1259 (14.3)
**Macro level variables**	
Insurance type	
*Statutory insurance*	4755 (54.1)
*Statutory + supplementary private insurance*	4031 (45.9)
Receive supplementary government support	417 (4.8)

#### Meso level variables

Among our participants, 36.4% reported receiving daily informal support from another individual while 30.6% reported receiving daily support from one or more of the listed organizations. In terms of social support, 80.7% reported to have low to moderate support available (Table 2).

#### Macro level variables

Of the 8786 participants in our study, 45.9% reported to have statutory insurance coupled with a supplementary private insurance, and 4.8% reported to receive supplementary government support (Table 2).

Further detailed descriptive results can be found in Table 2, whereas more detailed results on the values of EQ-5D-index and EQ-VAS by level of each independent categorical variable (reported by the levels of the ecological model) can be found in Additional file 1: Table 1.

### Multivariate regression of factors associated with HRQoL by levels of the ecological model

#### Micro level factors

The Tobit regression showed that older age was associated with a lower HRQoL only for the EQ-5D-index. On the other hand, female gender was significantly associated with both a lower EQ-VAS and a lower EQ-5D-index. In addition, having a lower level of education was found to be associated with a lower HRQoL. More specifically, having a primary level education or no education as compared to higher education, was significantly associated with a lower EQ-VAS and EQ-5D-index. A higher individual income was significantly associated with a higher EQ-5D-index.

In terms of health status, having vision, hearing and memory problems in daily life, as well as taking more than four types of medications daily were significantly associated with a lower EQ-VAS and EQ-5D-index. The same was true for the three variables denoting socioemotional well-being (feeling empty, missing company of others and feeling abandoned), which were significantly associated with a lower HRQoL (Table 3).

**Table 3:** Results of Tobit multivariate regression by levels of the ecological model

Variable	EQ-VAS			EQ-5D-index		
	Coeff	95% CI		Coeff	95% CI	
*Micro level variables*						
Age	− 0.02	− 0.08	0.03	− 0.0006*	− 0.001	0.0001
Gender (Ref: Male)	− 1.2*	− 1.8	− 0.6	− 0.02*	− 0.025	− 0.015
Education (Ref: Tertiary)						
Secondary	0.2	− 0.4	0.9	− 0.009	− 0.006	0.004
Primary or none	− 1.2*	− 0.2	− 2.1	− 0.01*	− 0.002	− 0.018
Other	0.7	− 0.7	2.2	− 0.001	− 0.001	0.026
Individual Income	− 0.008	− 0.0001	0.0001	0.0065*	0.006	0.007
Living situation (Ref: Living alone)	0.05	− 0.25	1.3	0.001	− 0.007	0.005
Vision problems (Ref: No)	− 4.6*	− 5.7	− 3.5	− 0.04*	− 0.05	− 0.03
Hearing problems (Ref: No)	− 3.0*	− 3.9	− 2.2	− 0.02*	− 0.02	− 0.01
Memory problems (Ref: No)	− 3.3*	− 4.0	− 2.5	− 0.02*	− 0.03	− 0.01
Unintentional weight loss (Ref: No)	− 7.9*	− 9.4	− 6.5	− 0.05*	− 0.06	− 0.04
Polypharmacy (Ref: No)	− 8.7*	− 9.3	− 8.1	− 0.05*	− 0.06	− 0.04
Feel empty (Ref: Do not feel empty)	− 4.6*	− 5.4	− 3.8	− 0.05*	− 0.057	− 0.044
Missing company of others (Ref: Do not miss company of others)	− 1.4*	− 2.1	− 0.8	− 0.010*	− 0.015	− 0.004
Feel abandoned (Ref: Do not feel abandoned)	− 2.9*	− 3.9	− 1.8	− 0.049*	− 0.057	− 0.040
Physical activity (Ref: Per WHO recommendations)	0.2	− 0.5	0.9	− 0.013	− 0.007	0.004
Alcohol intake (Ref: ≤ 2 alcoholic drinks/days)	0.5	− 0.6	1.6	0.003	− 0.006	0.012
Smoking (Ref: No)	0.2	− 0.9	1.3	0.007	− 0.001	0.017
*Meso level factors*						
Receive support from others (Ref: Support not needed)	0.02	− 0.4	0.9	− 0.0008	− 0.006	0.005
Receive support from organizations (Ref: No)	− 0.002	− 0.6	0.6	− 0.004	− 0.009	0.0007
Availability of social support (Ref: Very high to high)	1.4*	0.6	2.1	0.034*	0.027	0.04
Social activities (Ref: Active in more than one activity)						
Active in one activity	− 2.5*	− 3.1	− 1.9	− 0.02*	− 0.028	− 0.018
Not currently active/wish to be	− 4.5*	− 5.4	− 3.6	− 0.04*	− 0.048	− 0.033
*Macro level factors*						
Insurance type (Reference: Statutory insurance)	1.1*	0.5	1.7	0.005*	0.0003	0.01
Suppl. government insurance (Ref: No)	− 0.4	− 1.8	0.8	0.004	− 0.007	0.012
Adjusted R-Squared	0.2354			0.2402		

*p < 0.05

#### Meso level factors

Availability of social support and participation in social activities were significantly associated with both EQ-VAS and EQ-5D-index. More specifically, participants who reported to have a lower level of social support available, had a higher EQ-VAS and EQ-5D-index. Furthermore, participants who reported to engage in only one of the social activities listed had a significantly lower EQ-VAS and EQ-5D-index. The same was also true for participants who reported to engage in none of the social activities listed, who also had a significantly lower EQ-VAS and EQ-5D-index (Table 3).

#### Macro level factors

The Tobit linear regression revealed that having a supplementary private insurance in addition to statutory insurance was significantly associated with a higher HRQoL, for both EQ-VAS and EQ-5D-index (Table 3).

## Discussion

In this cross-sectional population survey conducted in one Swiss Canton, we assessed the overall HRQoL and factors associated with it among home-dwelling older adults aged 75 and above. These factors were organized into levels of the ecological model to account for the multidimensional nature of this construct. The mean EQ-VAS values in our study were slightly higher but similar to findings of König et al., who compared the HRQoL of older adults in six European countries using the same measurement tool as our study [57]. Our results also corroborate those of another national Swiss survey of community-dwelling older adults, where the mean EQ-VAS scale score was reported to be similar to our findings [29].Moreover, the distribution of frequencies and proportions by dimension and level of the EQ-5D-5L instrument were also similar between our study and the one of Luthy et. al. [29].

The findings of our study provide a more comprehensive understanding of factors that play a role in how older adults perceive their HRQoL and provide insight into which modifiable factors could be targeted to improve HRQoL in this population. We found that being privately insured was associated with a better HRQoL. We assume that in Switzerland, having supplementary private insurance is positively correlated with higher financial resources because while everybody is insured with the statutory insurance, supplementary insurance is typically only purchased by those who can afford the schemes. This is also in line with our results and findings of several other studies from countries with an aging population similar to Switzerland, which found a significant association between higher income and better HRQoL [58, 59].

Another important sociodemographic factor that was associated with HRQoL was level of education. Having a better education, which is a factor that is typically defined in younger stages of the life course, was linked to a better HRQoL among older adults. This finding corroborates with findings from studies in other countries with different cultures [60–62], pointing to the widespread influence of education, as well as of income, as important social correlates of health and HRQoL. Having a better education has been previously linked to higher health literacy which has been also shown to be linked to better HRQoL [63]. However, because we have not measured health literacy specifically, we refrained from assuming such an association.

From the literature, we had expected that having more availability of social support would be associated with a higher HRQoL among older adults [64, 65]. Interestingly, we observed the opposite among participants in our study, where receiving a low to moderate (tangible and emotional) social support was associated with a better HRQoL. We detected that the majority of participants had reported the availability of social support to be low to moderate. This might indicate that these participants do not require as much social support and might be more independent in the first place, thus consequently also reporting a better HRQoL. In addition to social support, being active, especially in more than one social activity, was found to be associated with a better HRQoL [66, 67].

Concerning health status and polypharmacy, our findings support those from the current literature on older adults. Self-reported hearing difficulties were consistently found to be associated with a lower HRQoL [22, 68, 69], and the same has been reported for visual impairment [70–72] as well as polypharmacy [73, 74]. In line with physical well-being, our analysis also revealed that socioemotional well-being played an important role in how older adults perceived their HRQoL. Feelings of emptiness and abandonment, along with missing the company of other people were all found to be negatively associated with HRQoL, findings which are also substantiated by other researchers [75, 76].

In line with other research findings, we also found that being female and older was associated with a poorer HRQoL [60, 77]. However, in our study we found that age was significantly associated with HRQoL as measured by the EQ-5D-index but not the EQ-VAS. This difference in significance can be explained by the different methodological measurements applied for these two constructs: EQ-5D-index is based on standard value sets whereas the EQ-VAS is based on the self-rating of our participants. We used the German value sets in lack of Swiss ones, based on the general recommendations to select a value set based on geographic proximity [78]. A Swiss study on cancer patients compared the use of both German and French value sets, as two countries sharing the geographical border with Switzerland, and found that the French value sets were more appropriate for this population [79]. Nevertheless, due to the fact that German language is spoken in the region of our research and considering our study was conducted in community-dwelling older adults, we believe our methodological choice was appropriate.

Based on the presented results, we reflect upon the fact that there might be a proportion of home-dwelling older adults who are living at a socio-economic disadvantage. Having fewer financial resources and a lower level of education, coupled with the presence of multimorbidity and loneliness, may contribute to deepening the disparities amid this population. Improving access to financial and social resources that facilitate a better standard of living can influence older adults' HRQoL and can potentially impact their ability to remain independent and age within their own familiar environments. To the best of our knowledge, this is the first Swiss study that uses an ecological approach based on the notion that HRQoL is a multidimensional concept and that in addition to health and social well-being, the environment plays an important role in how older adults perceive their HRQoL. While the outcome variable of our study implies an individual perception, HRQoL is a construct that is influenced by factors beyond the individual and thus further research studying a wider range of meso and macro variables such as for instance housing, age-friendly neighbourhoods and improved access to social activities is necessary.

We emphasise the importance of social determinants of health and potential disparities that exist, suggesting policymakers ought to focus on policies to reduce socio-economic inequalities. The impact of social determinants of health among the older population are a result of inequities from early stages of life and might not always be modifiable at a later stage in life, such as for example access to education or employment opportunities. However, and ideally, policymakers should focus on policies to reduce disparities considering a life-course approach, which could ultimately impact HRQoL in later stages of life. The social determinants of health are typically seen as being accountable for health inequities and can play an important role in the ageing trajectory of an individual and how they perceive their HRQoL. Therefore, exploring elements such as socioeconomic status, education, the physical environment, employment, and social support networks through an ecological lens like we proposed, can provide a deeper understanding of which factors influence the self-reported HRQoL. Our results also highlight that many of the identified factors are modifiable correlates of HRQoL, and provide public health indications that could support concrete actions. For example, investing in improving social networks and activities of older adults, which could help reduce loneliness or feelings of abandonment, could not only potentially improve their HRQoL, but also aid them in maintaining the desired independence to continue living longer within their communities.

### Strength and limitations

We consider the population survey methodology to be a considerable strength of our study because it provides a representative sample of the population we targeted. We achieved an overall response rate of 30.7% which is considerably higher than the average response rate found in other population surveys using postal delivery modes [40]. This response rate is also particular given that our target population has been known to be challenging to reach and might have needed additional support to fill out the questionnaire [80]. Furthermore, we believe that using the ecological approach has provided a more comprehensive lens on the HRQoL of older adults, by placing them at the center of their ecosystem.

The present study does however come with certain limitations that we acknowledge. It is possible that older adults who responded to our survey might have been healthier and more engaged in social life compared to their older, frailer or cognitively challenged counterparts who did not respond, thus subjecting our study to potential selection bias. Furthermore, this study was conducted among home-dwelling older adults, excluding an important segment of the older population who reside in long-term care facilities. As of 2017, the proportion of the population aged 80 years or older in Switzerland that resides in a nursing home is around 15% [81], meaning our study could not capture the HRQoL and factors correlated with it in this portion of the population. Moreover, we conducted our research in only one Swiss Canton, whose language and socio-cultural aspects might make it unique and distinguishable from other regions and accordingly might limit the generalizability of our results. Furthermore, the cross-sectional nature of the design also limits us in inferring any direct causal link between the variables and HRQoL. Finally, although a plethora of micro level factors was available for analysis, we were limited in the variables available on the meso and macro level.

## Conclusions

Understanding factors related to HRQoL by using an ecological lens can help identify factors beyond the individual level that impact the HRQoL of home-dwelling older adults. Our study emphasises the importance of social determinants of health and potential disparities that exists, encouraging policymakers to focus on policies to reduce socio-economic disparities and support interventions that take social factors into account. We anticipate that this study helps to increase awareness that HRQoL in older adults is multidimensional and thus multifaceted interventions that try to interrelate health services, social services and environmental factors are needed.

## Supplementary Information

**Additional file 1. Supplementary Table 1: **Values of EQ-5D-index and EQ-VAS by level of each independent categorical variable (reported by the levels of the ecological model).

## Data Availability

The dataset generated and/or analysed during the current study is not publicly available as its participants belong to a vulnerable population, but is available from the corresponding author on reasonable request.

## Abbreviations

Canton BL: Canton Basel-Landschaft
QoL: Quality of life
HRQoL: Health-related quality of life
VAS: Visual analogue scale
GFI: Groningen Frailty Index
BS6: Brief Social Support Scale
VIF: Variance Inflation Factor
MAR: Missing at random
MCAR: Missing completely at random
