# Supplementary material for: Factors associated with health-related quality of life among home-dwelling older adults aged 75 or older in Switzerland: a cross-sectional study
Source: Health Qual Life Outcomes. 2022 Dec 21;20:166. doi: 10.1186/s12955-022-02080-z (PMC9773624; doi:10.1186/s12955-022-02080-z)
Supplement: Supplementary file 1 — Additional file 1. Supplementary Table 1: Values of EQ-5D-index and EQ-VAS by level of each independent categorical variable (reported by the levels of the ecological model). [file 12955_2022_2080_MOESM1_ESM.docx]

Supplementary Table 1: Values of EQ-5D-index and EQ-VAS by level of each independent categorical variable (reported by the levels of the ecological model)

| **Variables** | **EQ-VAS (mean, SD)** | **EQ-index (mean, SD)** |
| --- | --- | --- |
| **Micro level variables** | | |
| Gender |  |  |
| *Male* | **76.1 (15.2)** | **0.91 (0.12)** |
| *Female* | **74.5 (16.4)** | **0.88 (0.14)** |
| Education |  |  |
| *Tertiary* | 74.8 (15.9) | **0.89 (0.12)** |
| *Secondary* | 75.2 (16.0) | **0.89 (0.14)** |
| *Primary / No education* | 76.1 (15.3) | **0.91 (0.12)** |
| *Other* | 75.5 (15.6) | **0.91 (0.10)** |
| Living situation |  |  |
| *Living alone* | 75.0 (15.9) | 0.90 (0.13) |
| *Living with others* | 75.4 (15.8) | 0.90 (0.13) |
| Vision problems |  |  |
| *No* | **76.2 (15.3)** | **0.90 (0.12)** |
| *Yes* | **65.5 (18.0)** | **0.81 (0.20)** |
| Hearing problems |  |  |
| *No* | **76.5 (15.3)** | **0.91 (0.12)** |
| *Yes* | **69.4 (17.0)** | **0.85 (0.16)** |
| Memory problems |  |  |
| *No* | **76.7 (15.2)** | **0.91 (0.11)** |
| *Yes* | **69.2 (17.2)** | **0.85 (0.17)** |
| Unintentional weight loss in past 6 months |  |  |
| *No* | **75.8 (15.4)** | **0.90 (0.12)** |
| *Yes* | **62.0 (18.8)** | **0.80 (0.22)** |
| Polypharmacy |  |  |
| *No* | **80.5 (13.1)** | **0.93 (0.08)** |
| *Yes* | **69.4 (16.6)** | **0.86 (0.16)** |
| Feeling empty / sometimes feeling empty |  |  |
| *No* | **77.6 (14.6)** | **0.92 (0.10)** |
| *Yes* | **67.6 (17.4)** | **0.82 (0.18)** |
| Miss company / sometimes miss company |  |  |
| *No* | **77.6 (15.1)** | **0.92 (0.11)** |
| *Yes* | **71.0 (16.4)** | **0.86 (0.16)** |
| Feeling abandoned / sometimes feeling abandoned |  |  |
| *No* | **76.2 (15.3)** | **0.91 (0.11)** |
| *Yes* | **66.7 (17.8)** | **0.80 (0.20)** |
| Physical activity |  |  |
| *As per recommendations of WHO* | 75.4 (15.8) | 0.90 (0.13) |
| *Below the recommendations of WHO* | 74.8 (15.9) | 0.90 (0.13) |
| Alcohol intake |  |  |
| *≤2 drinks/day* | 75.1 (16.0) | **0.90 (0.13)** |
| *>2 drinks/day* | 76.7 (14.2) | **0.91 (0.12)** |
| Smoking |  |  |
| *No* | 75.2 (15.9) | 0.90 (0.13) |
| *Yes* | 75.2 (15.8) | 0.90 (0.12) |
| **Meso level variables** | | |
| Receive support from individuals |  |  |
| *No* | 75.3 (15.8) | 0.90 (0.13) |
| *Yes* | 75.1 (15.9) | 0.89 (0.13) |
| Receive support from organizations |  |  |
| *No* | 75.4 (15.6) | 0.90 (0.13) |
| *Yes* | 74.8 (16.3) | 0.89 (0.14) |
| Availability of social support |  |  |
| *High to very high* | **73.5 (17.8)** | **0.86 (0.18)** |
| *Low to moderate* | **75.6 (15.3)** | **0.90 (0.11)** |
| Social activities |  |  |
| *Active in more than one activity* | **77.9 (14.2)** | **0.92 (0.09)** |
| *Active in one activity* | **73.7 (16.2)** | **0.88 (0.14)** |
| *Not currently active / wish to be* | **69.6 (18.2)** | **0.85 (0.18)** |
| **Macro level variables** | | |
| Insurance type |  |  |
| *Statutory insurance* | **74.3 (16.4)** | **0.89 (0.14)** |
| *Statutory + supplementary private insurance* | **76.3 (15.1)** | **0.90 (0.12)** |
| Receive supplementary government support |  |  |
| *No* | 75.3 (15.8) | 0.90 (0.13) |
| *Yes* | 74.7 (16.1) | 0.89 (0.13) |

Note: Results in bold indicate a significant difference (p < 0.05) as revealed by the Tobit multiple

linear regression modelling
